# Joseph Carpue's file drawer experiment – A murder mystery from 1801

**DOI:** 10.1016/j.jpra.2015.10.005

**Published:** 2015-11-18

**Authors:** M. Felix Freshwater

**Affiliations:** Voluntary Professor of Surgery University of Miami School of Medicine, 9155 S Dadeland Blvd. Suite 1404, Miami, FL 33156-2739, USA

**Keywords:** History of medicine, Biography, File drawer experiment, Benamin West, Joseph Carpue

## Abstract

Today unpublished or “file drawer” experiments are the impetus for trial registration and reporting of all results. In 1801, Joseph Carpue, the father of modern plastic surgery, did a file drawer experiment for Benjamin West, who was President of the Royal Academy of Arts. George III had commissioned West to create the largest stained glass window ever created whose theme, the Crucifixion, was based upon Michelangelo's drawing. Subsequently, West suffered a series of political, professional and economic setbacks. In the summer of 1801, West's project was delayed. By the fall, West hoped that independent scientific confirmation of his design could salvage the project. West approached Carpue who obtained a murderer's fresh corpse that he crucified and documented the results with plaster casts created by sculptor Thomas Banks. Carpue's experiment showed that West's window design wrongly depicted the Crucifixion because West had posed the hands and shoulders incorrectly. West died in 1820 without ever being associated with Carpue's experiment. Carpue's obituary in The Lancet in 1846 contained Carpue's handwritten note that described the experiment but not West's Royal commission. As no records or publications associate the cast with West project, this can be considered to be a file drawer experiment. After 1801, West made further drawings of the Crucifixion that showed the figures in the same position as the cast. Nineteenth century auction catalogues suggest that West made a corrected Crucifixion painting, but its current location remains a mystery.

Many plastic surgeons consider themselves artists, but most are unaware that at the beginning of the 19th century a surgical experiment answered a centuries old art question. Today unpublished experiments are called “file drawer” experiments and are the impetus for trial registration and reporting of all results because concealment creates the false impression that published studies with positive results are valid.^1^ Experiments may be relegated to file drawers for a host of reasons, including concern by either the researchers or their sponsors that publication of the findings could yield unfavourable consequences.

Scientific research was suppressed hundreds of years ago. In the 17th century, the Inquisition tried and convicted Galileo for defending Copernicus's theory of heliocentrism that Galileo had verified via his own astronomical observations. However, file drawer experiments are not suppressed by external authorities; rather they are suppressed by those directly involved with the experiments. The earliest known clinical trial was that of Lind on the treatment of scurvy, which he published in 1753.^2^ While the ‘file drawer problem’ was not named in 1979, one might ask how soon after Lind's trial were other experiments buried and why?

The secrecy surrounding file drawer experiments makes it difficult to determine when the first file drawer experiment was done. This is the story of one such experiment done in 1801. In addition to having been a file drawer experiment, it was a murder mystery as it contained a murder, missing evidence and a potential scandal that if disclosed could have upset the balance of power in the British art establishment during early 19th century.

## The scientist: Joseph Constantine Carpue

**Figure. 1:**
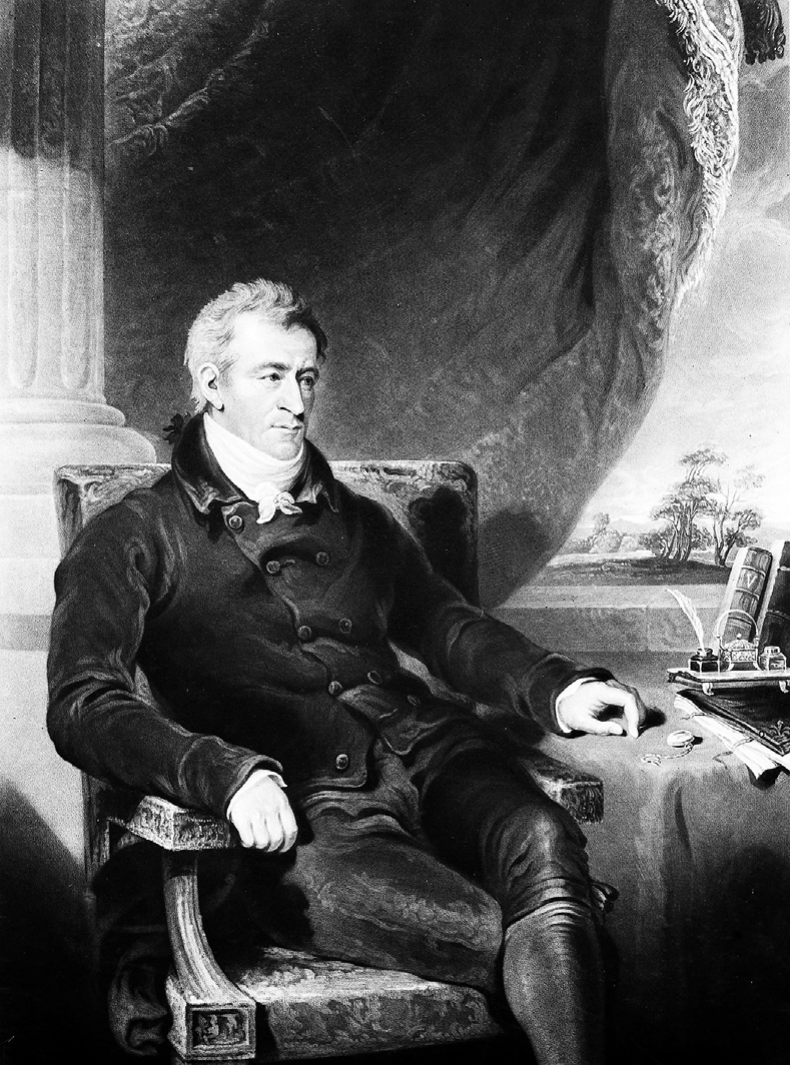
Joseph Constantine Carpue engraving by Charles Turner. Courtesy of the Wellcome Library, London. Turner was the foremost engraver in England whose engravings of J.M.W. Turner's paintings opened them to popular consumption.

**Figure. 2:**
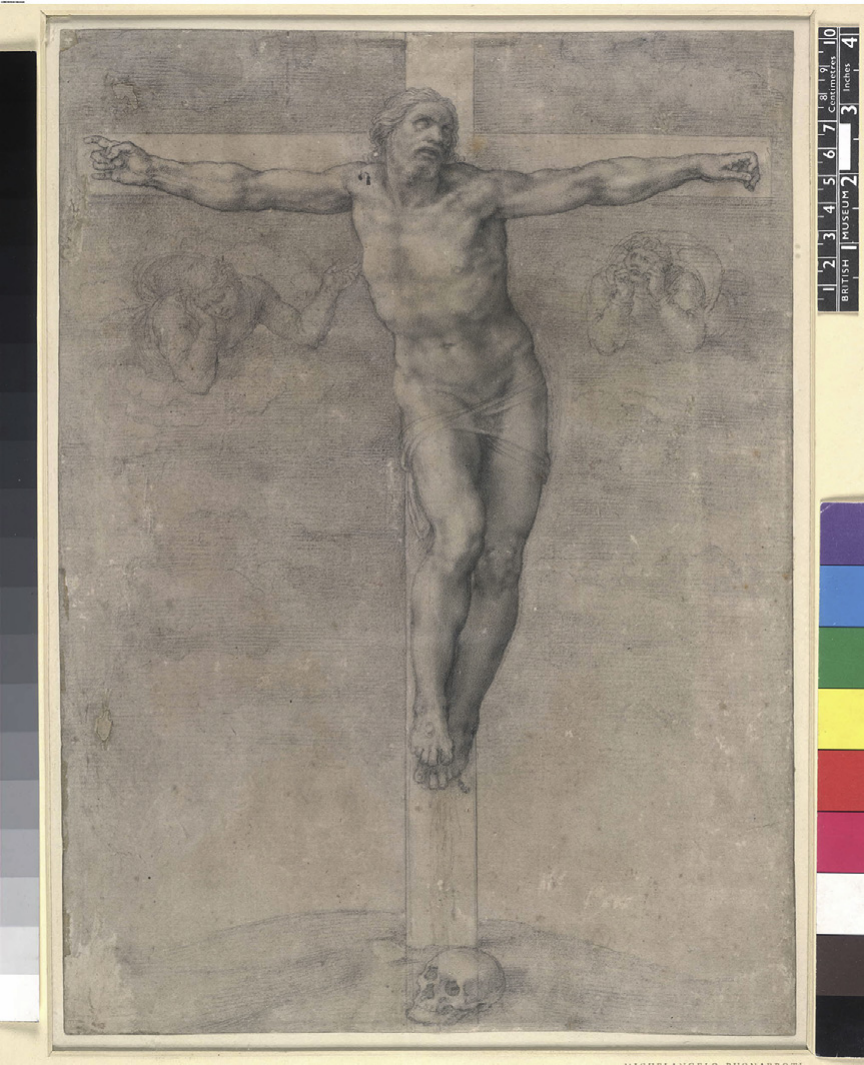
Crucifixion by Michelangelo, a drawing in black chalk 36.8 × 26.6 cm https://www.britishmuseum.org/explore/online_tours/europe/michelangelos_drawings/crucifixion_by_michelangelo,_a.aspx [Accessed July 21, 2015] (British Museum).

Joseph Carpue was an anatomist, teacher and surgeon who practiced in London in the first part of the 19th century (Figure 1). Today he is best known as the father of modern plastic surgery, but he also published monographs on the anatomy of muscles, experiments with galvanism and the technique of superpubic lithotomy.^3^, ^4^, ^5^, ^6^ A note found in his papers was quoted in this 1846 obituary:

“Some time in the year 1800 … Mr. West, President of the Royal Society (sic), Mr. Banks, and Mr. Cosway … having agreed amongst themselves that the representation of the Crucifixion did not appear natural, though it had been painted by the greatest artist of his age, wished to put this to a test (Figure 2) … They, therefore, requested me to nail a subject to a cross … Shortly after this application, a circumstance occurred at … Chelsea, which enabled me to comply with their request. A man of the last name of Legg, one of the captains of the hospital, having had a dispute with a man named Lamb … Legg fired [a] pistol, and shot Lamb through the thorax. He immediately expired. I was at the time surgeon of Chelsea … a jury sat on the body; the verdict was wilful (sic) murder. . Mr. Keate, surgeon general and surgeon of the hospital, was master of the College of Surgeons; to him I applied for the body when executed … He promised to give the sheriff an order that the subject might be given for the purpose required. A building was erected near the place of execution; a cross provided; the subject was nailed on the cross; the cross suspended; when the body being warm, fell into the position that a dead body must fall into, let he cause of death be what it may. When cool, a cast was made, under the direction of Mr. Banks, and when the mob had dispersed, it was removed to my theatre … The cast is still in existence, and is preserved in the studio of Mr. Behnes.”^7^

Carpue's account was written some time after the events as some of his facts were contradicted by contemporaneous sources. The murder, trial, execution and experiment occurred in 1801, not 1800. Legg shot Lamb on October 2, 1801, was tried on October 28, 1801, and was executed five days later.^8^, ^9^ Benjamin West was the President of the Royal Academy of Arts, not the Royal Society. Nevertheless, Carpue's account described two vital elements of his experiment, first, the hypothesis that the Crucifixion had not been accurately depicted in many works of art and, second, that the method used to test the hypothesis and record the results was crucifixion followed by casting. Left unsaid by Carpue was the Royal Academicians' motivation for testing the hypothesis and what would have been the consequences of accepting or rejecting the hypothesis.

I believe that other contemporaneous facts suggested that what Carpue described was an early example of a file drawer experiment and that the result was suppressed because had it been exposed then it could have caused professional and financial damage to West. To understand why, we must examine the state of the Royal Academy and West, its leader, at the end of the 18th century.

## The sponsor: Benjamin West

**Figure. 3:**
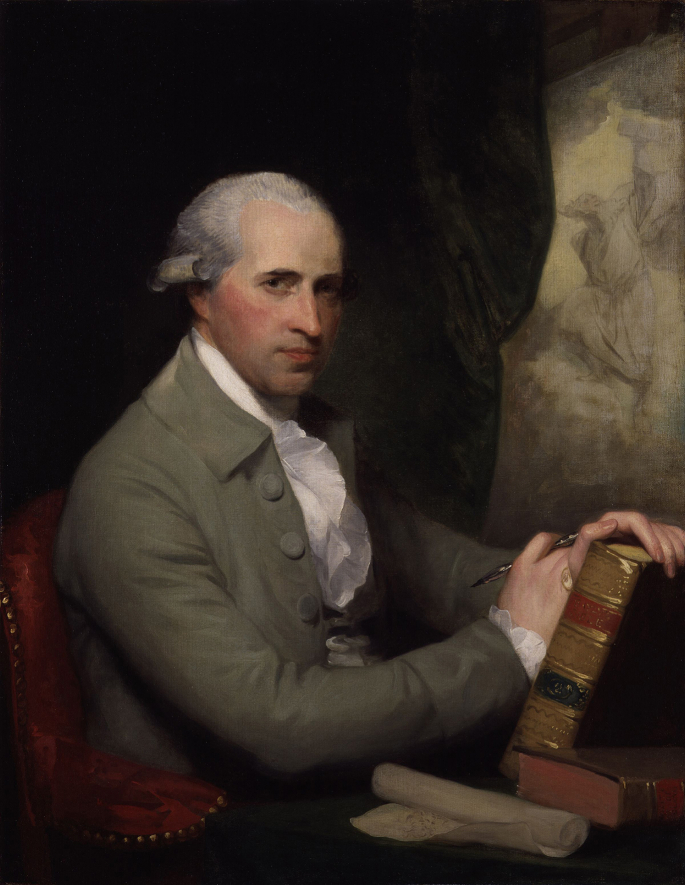
Benjamin West by Gilbert Stuart, Oil on canvas, 1783–84 35 1/2 × 27 1/2 in. (90.2 × 69.9 cm) Smithsonian National Portrait Gallery, Washington. Stuart was a student of West's at the Royal Academy. He became a portraitist who is best known for his portrait of George Washington.

Benjamin West was a master painter, politician and opportunist (Figure 3). Born in Pennsylvania in 1738, he travelled to Italy in 1760 and in 1762 met Richard Dalton, who was George III's librarian and keeper of pictures. Dalton was George III's agent and made major art acquisitions for him. Impressed by Wests' ability, Dalton commissioned a painting, *Cimon and Iphigenia*, for George III and suggested that West go to London after leaving Italy.^10^ West arrived in London in 1763. Shortly thereafter, George III gave West his first commission and over the ensuing years, they spent much time discussing how to raise interest in the fine arts in England. Encouraged by West, George III established the Royal Academy of Arts in 1768. By 1772, West had been appointed historical painter to the court with an annual stipend of £1000. Eventually, he earned over £30,600 from royal commissions. This is approximately £35,110,000 of income in 2013.^11^

**Figure. 4:**
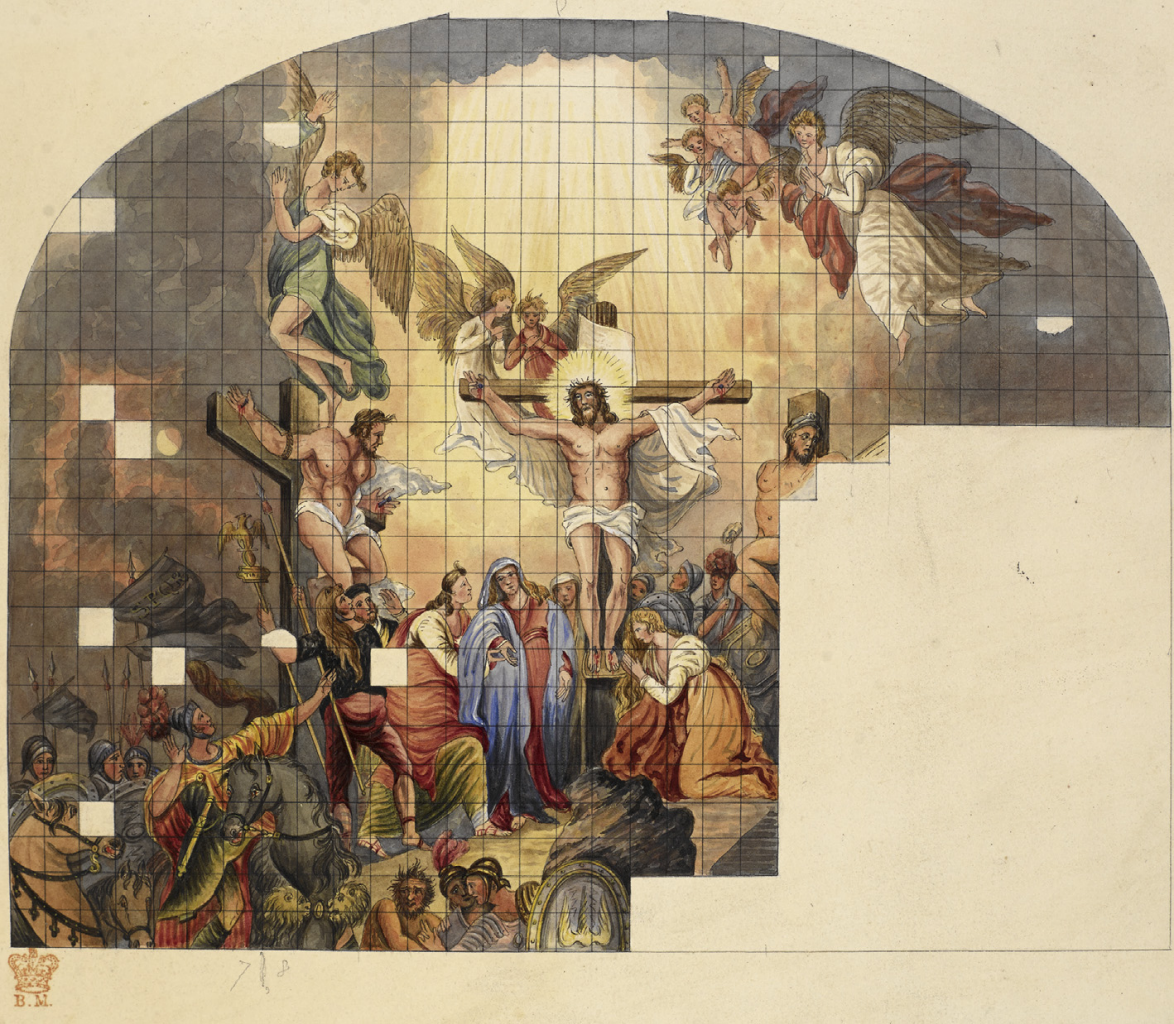
Sketch of an unfinished Window painted on Glass by Mr. Forrest from a Cartoon by Benj. West intended for the Great West Window of St. George's Chapel, Windsor. Willement T. Collection of drawings, etc., partly by his own hand, of ancient and modern stained glass, ecclesiastical and domestic, chiefly in Great Britain and France. 1844; Add. MS 34873, no. 47, f. 17. (British Library) Note the extended digits and shoulders in 90° abduction similar to Figure 2.

In 1784, George III acceded to West's proposal to redesign St. George's Chapel at Windsor and install new art and stained glass windows. West's designs included giant stained glass windows depicting the Resurrection and Crucifixion. The latter was the largest stained glass window created to that time as it measured 28 by 36 ft. Including fabrication, the window cost £2100 (Figure 4).^12^ By 1792, West had reached the pinnacle of the art establishment after he had succeeded Sir Joshua Reynolds as president of the Royal Academy. At the time, it was said that George III had offered West a knighthood, but that he had refused it and hinted that he would accept a baronetcy.^13^

## The Venetian scandal

**Figure. 5:**
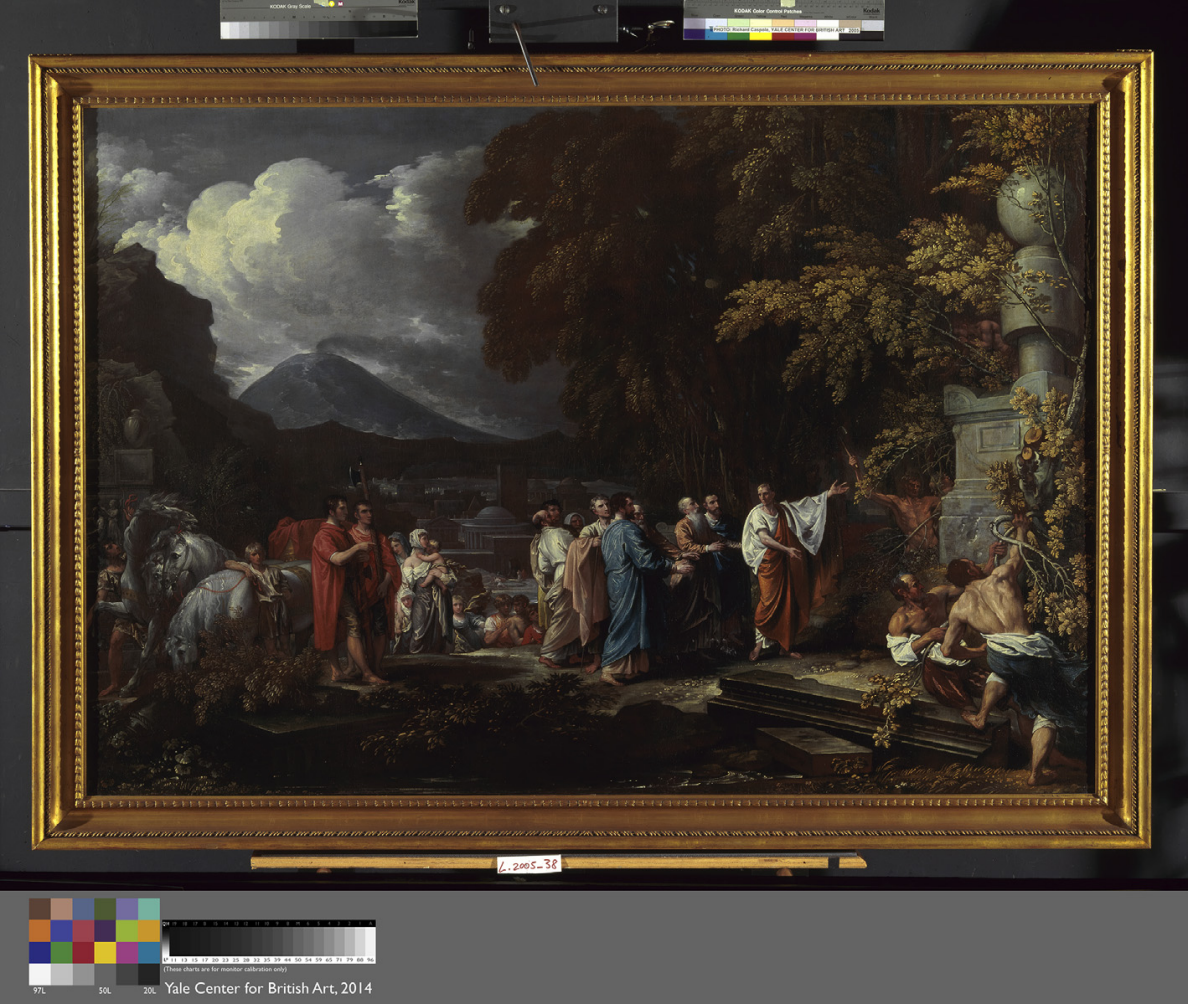
Cicero Discovering the Tomb of Archimedes by Benjamin West, Oil on canvas, 1796, 124.5 × 180.5 cm (A private collection) Note how the scene appears to have occurred at night which was caused by West's use of the Plovis' secret formula.

West and other Royal Academicians were enraptured by the art of high Renaissance painters such as Titian. They reasoned that if only they had Titian's materials and techniques, then they could paint like Titian. Blinded by their envy, beginning in 1795 West led a group of academicians who were defrauded by a father and daughter named Plovis who claimed to have inherited Titian's materials and techniques. The Plovis' required that knowledge of Titian's materials and techniques be limited to only those academicians who had purchased a share from them and signed a secret nondisclosure agreement. West, the wily politician, never created a paper trail that could have associated him with the Plovis'. He neither purchased a share nor signed the nondisclosure agreement, yet, he used the Plovis' formula to create at least two paintings that he displayed at the 1797 Academy show “Cicero Discovering the Tomb of Archimedes” (Figure 5) and an oil painting, upon which he based his St. George's Chapel “Crucifixion”.^14^ Contemporary art critics who viewed the paintings were scathing in their remarks. Some said that the lighting in Cicero was so dark that the scene appeared to have occurred at night. While the extant libel laws limited what critics could write, caricaturists had artistic freedom that allowed them to be even more ruthless than critics. In 1797, James Gillray indicted the folly of West, other Royal Academicians and the press in “Titianus Redivivus; or the Seven Wise men Consulting the new Venetian Oracle – a Scene in ye Academic Grove.” West is the rightmost figure in the print and, as noted by his balloon, is attempting to distance himself from the fiasco: “Charming secret friend for thee to dash out another gallery with — but I'm off!!”^15^ (Figure 3).

## 1801 – West's *annus horribilis*

By 1801, West still had not recovered from the embarrassment of the Venetian Secret. His personal and professional fortunes continued to wane. When governing the Royal Academy, he had ignored the bylaws regarding how members were elected to council and this authoritarian manner resulted in George III annulling the 1800 election results.^16^ West had cash flow problems from poor investments and extended family obligations.^17^ His relations with George III had deteriorated; the latter had ceased patronizing him, had been late in paying his stipend and had ordered him to suspend all work at Windsor that summer.^18^

## The file drawer experiment

As West had created the largest representation of the Crucifixion and, as Carpue suggested in his note, its anatomical accuracy was debated, West could have thought that he would improve his reputation, solidify his leadership position at the Royal Academy and be restored to George III's good graces if he had scientific proof that his representation of the Crucifixion was anatomically accurate. Why was West accompanied by Thomas Banks and Richard Cosway when approached Carpue and why did he choose Carpue?

West was the only one of the three who had created religious or historical themed works. Banks was a renowned sculptor who knew how to make plaster casts while Cosway was a portraitist, with no interest in religious paintings, but he was someone who had credibility, as he was one of the academicians not fooled by the Venetian Secret. Carpue was a suitable scientist for conducting such an experiment as he just had published his book on the anatomy of muscles and, through his relationship with Thomas Keate, had access to cadavers. The experiment's outcome was riskless for West. If it confirmed his anatomic representation, then he could announce the result and further rehabilitate his professional reputation that had been sullied by the Venetian Secret, but if the outcome contradicted his Crucifixion painting, then, as there was no paper trail, he could disavow any knowledge of the experiment and avoid his own reputational crucifixion.

In 1802, Thomas Banks asked the Royal Academy's Council to reimburse him for his costs. In describing the experiment, he never mentioned West:

“Gentlemen,

The figures on the crosses which I sent to the Royal Academy were moulded on the body of a Malefactor executed for the murder & ordered for dissection – the body was given by Mr. Keate Surgeon general to the army to Mr. Cosway, who with my self attended the operation of moulding casting and dissecting it, which dissecting was carefully & intelligently done by Mr. Carpue Surgeon and Anatomist of Leicesterfields; Mr Cosway my self and other members of the Royal Academy being of the opinion that such casts might be useful to the students of the Royal Academy & also to the Professor of Anatomy at the time of his giving lectures as the may be mov'd from the Antique Academy to the Lecture room and back again with very little trouble –

With this view principally they were done & sent & plac'd were they are for the approbation of the Council – the whole of the expense incur'd in moulding casting & mounting them does not exceed the sum of sixteen or eighteen pounds the particulars of which sum, I can furnish the Council with if they chuse to retain them, if not they shall be remov'd.”^19^

**Figure. 7:**
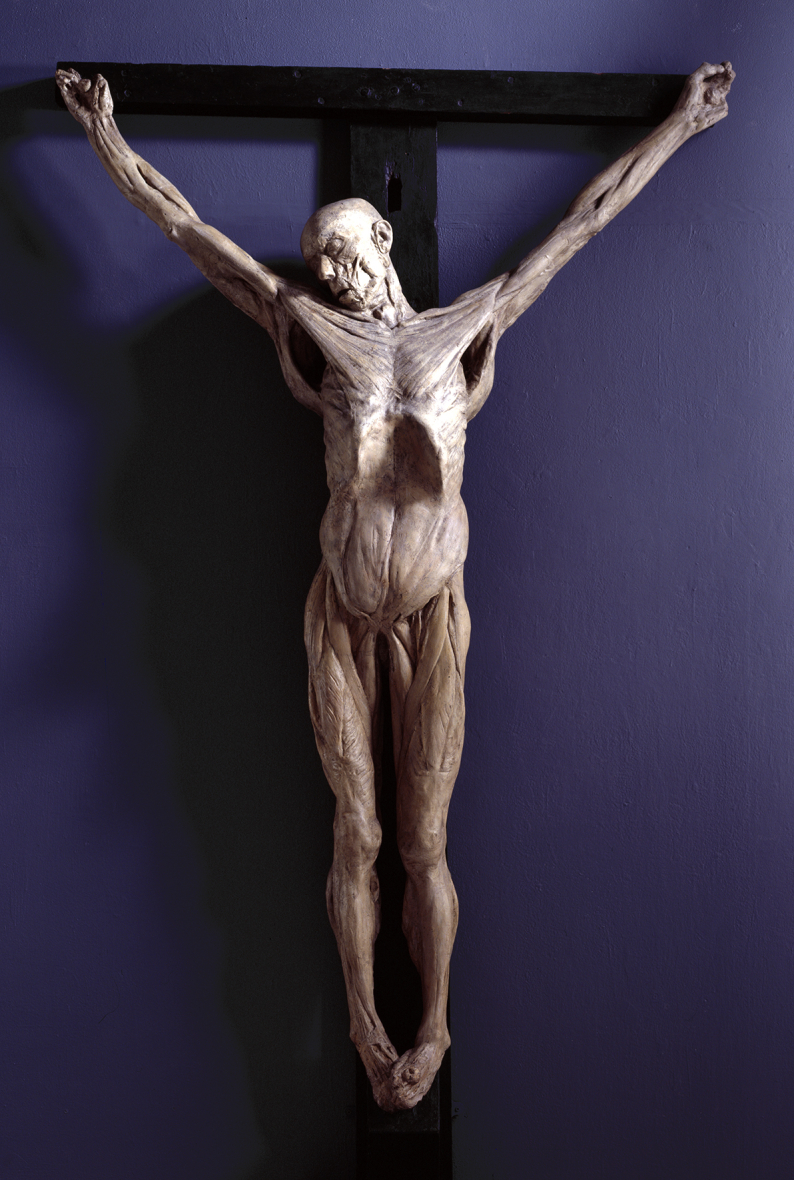
Anatomical Crucifixion (James Legg) by Thomas Banks, Plaster cast, 1801, 2315 × 1410 × 340 mm (Royal Academy of Arts). Note the remaining flexed digits the others broke over time.

The fact that Banks excluded West's name from this letter adds additional support to the theory that West purposely distanced himself from the experiment and even was absent from the site where Carpue did it. Indeed, the only known record of West's knowledge of the experiment was in *The Art Union*, a magazine published 25 years after his death for which Carpue served as the major source of information. In that article, after seeing the cast, West was purported to have said that, “He had never before *seen the human hand*.”^20^ Examination of the cast clearly demonstrates that West's design for the Crucifixion was wrong as none of the figures in West's design was drawn with flexed digits and shoulders abducted 135°. Instead, they were drawn with the digits extended and the shoulders abducted 90° (Figure 7).

While West's design was wrong, his error would become moot. Fortunately for West, forces beyond his control quashed the completion of his renovation of St. George's Chapel. George III had become ill and Forrest, the glassmaker, had died. The project was abandoned in 1808, the extant glass was placed in storage until 1846 when it was moved to Calcutta, installed in the new Anglican cathedral and destroyed by a cyclone in 1864.^21^

## West's atonements

**Figure. 8:**
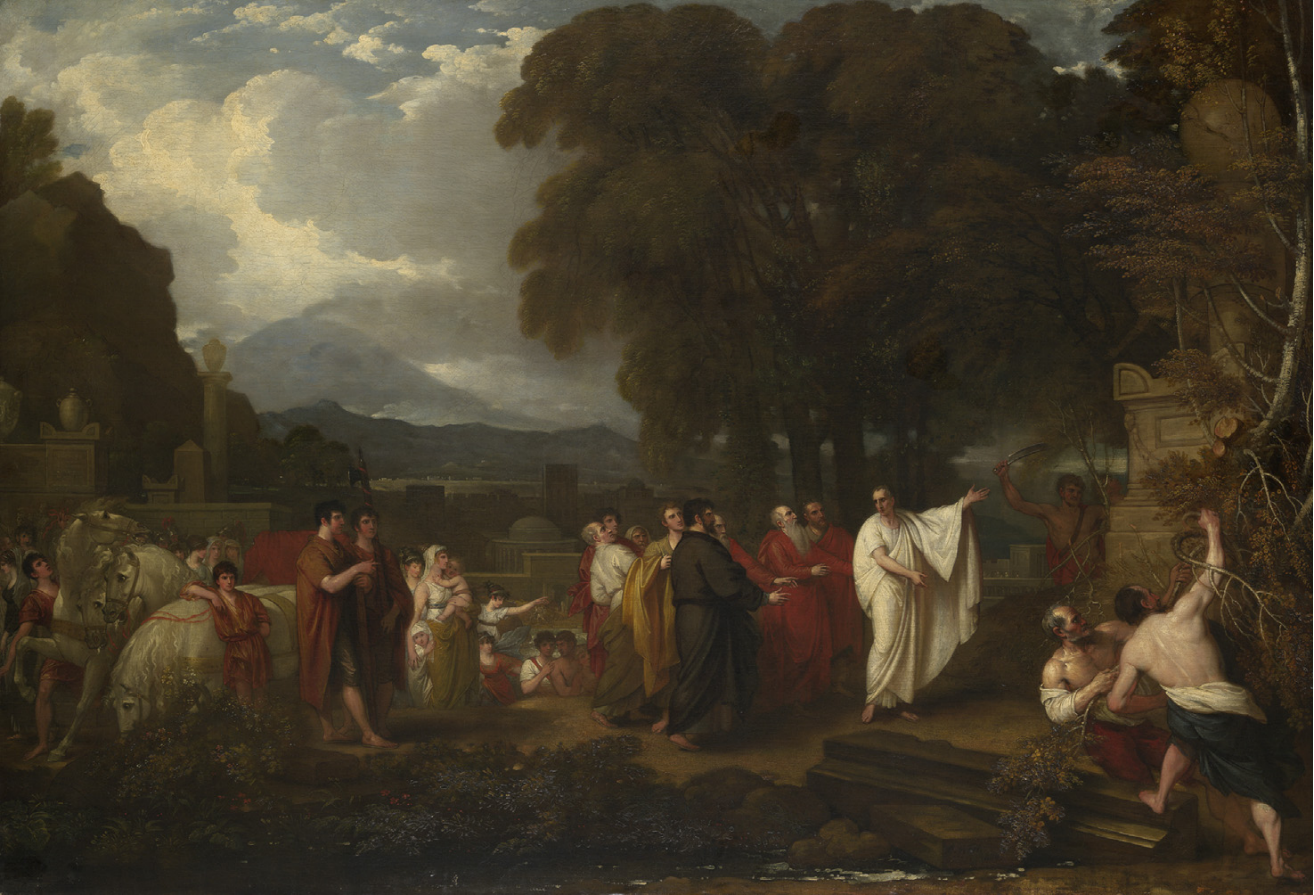
Cicero Discovering the Tomb of Archimedes by Benjamin West, Oil on canvas, 1804, 125.7 × 182.25 cm, Yale University Art Gallery. Art historians have called this painting West's atonement for his 1796 Cicero. Note how the lighting and composition differ from Figure 5.

Seven years after his public humiliation in the Venetian affair, West made a second painting “*Cicero Discovering the Tomb of Archimedes*”, but this time he did not use the Plovis' formulae. Although his reasons for creating a second *Cicero* are unknown, art historians who have analyzed both paintings have termed the second painting West's “Atonement” suggesting that an element of penance contributed to West's decision (see Figure 8)..^22^

**Figure. 9:**
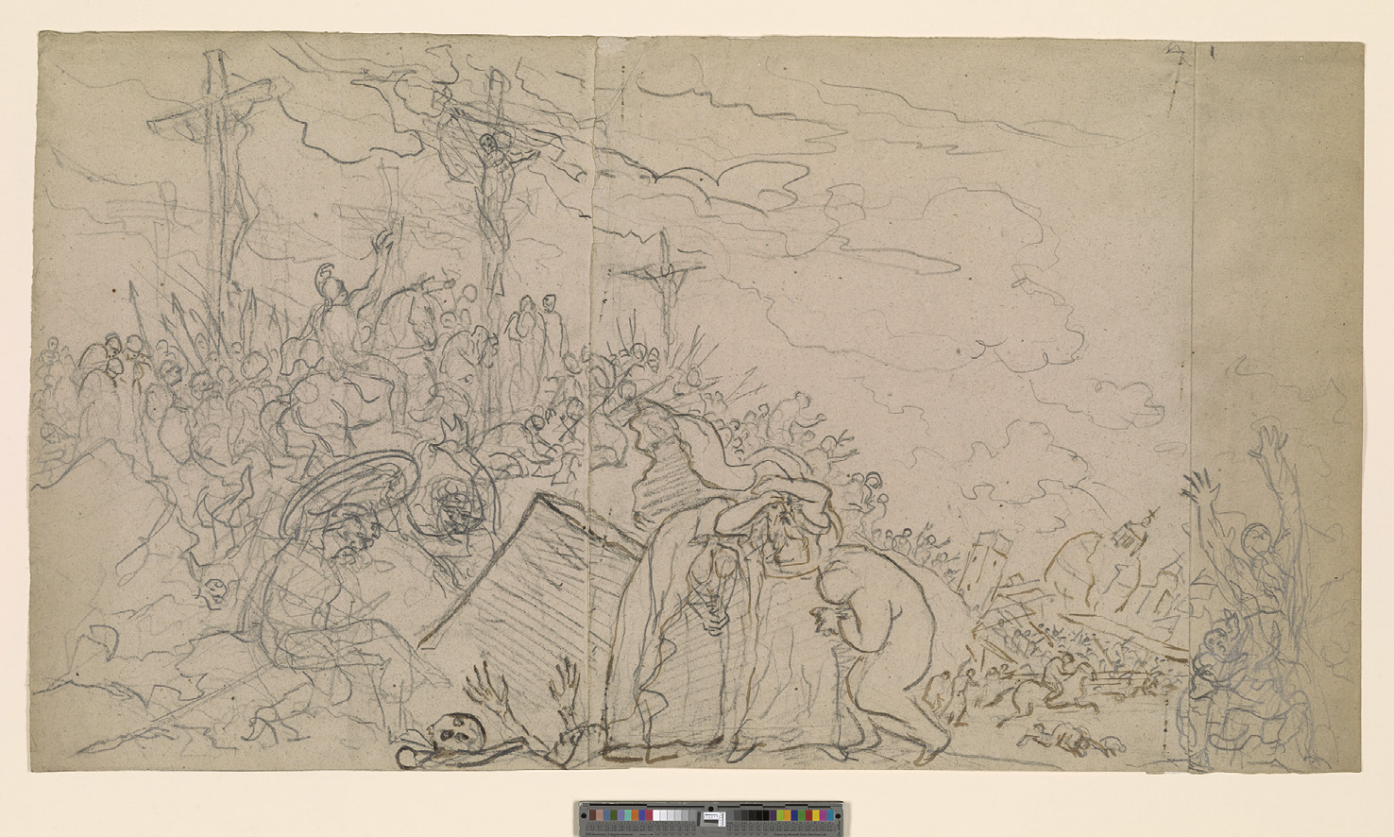
The Crucifixion by Benjamin West, black chalk with pen and brown ink, on three pieces of oatmeal paper originally pinned together by the artist, 1814, 32.5 × 58.5 cm (The Pierpont Morgan Library, New York. 1970.11:56. Purchased as a gift of Mrs. Robert H. Charles.) Note how the crucified figures position and perspective differ from Figure 4.

**Figure. 10:**
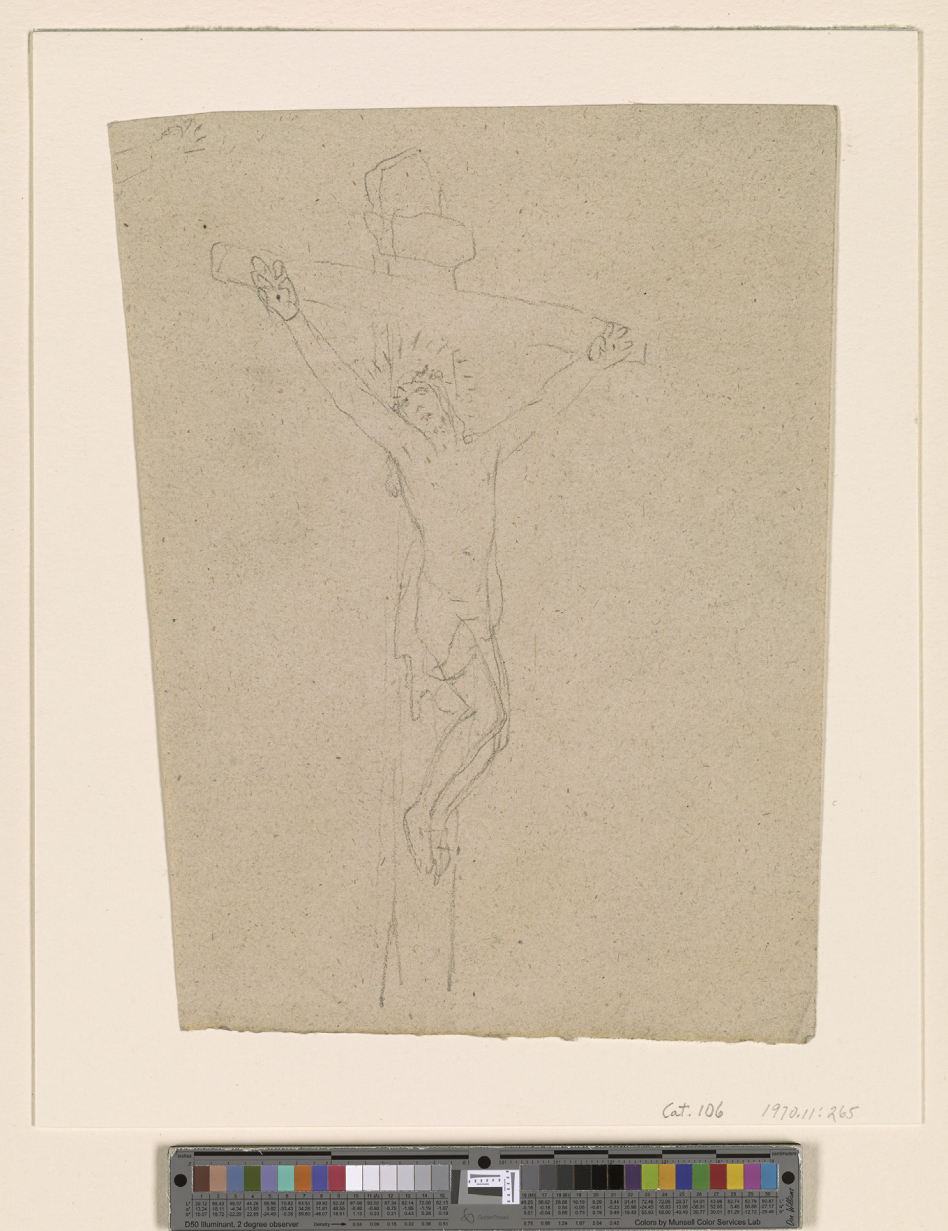
Christ on the Cross by Benjamin West, black chalk on oatmeal paper, 17- -, 32.5 × 58.5 cm (The Pierpont Morgan Library, New York. 1970.11:265. Purchased as a gift of Mrs. Robert H. Charles.) Note the flexed digits that differ from Figure 4.

West also atoned for his Crucifixion error. After Carpue's experiment, West created two drawings now owned by the Morgan Library in New York. Note how West reworked his Crucifixion window to match Legg's cast with Jesus' and the thieves' shoulders abducted to 135° (Figure 9). Note how the digits are flexed as they are in Legg's cast, which stresses the importance of West's stating that until he had seen the cast “He had never before *seen the human hand*” (Figure 10).

## The mystery of the missing paintings

Galt's biography of West was written with the latter's approval and contained a catalogue raisonné of his works that listed two Crucifixion paintings. Galt described the first as the model for the chapel window.^23^ In 1829, that painting was sold at auction, the catalogue stated that its dimensions were 7′6″ high and 6′ wide and described it as:

“Painted by command of His late Majesty. From this design a painted glass was to be executed for the large west window in St. George's Chapel, at Windsor.”^24^

Galt's description of the other Crucifixion painting was limited to its dimensions of 16 by 28 feet. Two facts suggest that this second Crucifixion painting was based upon the Morgan drawings. First, it is in a landscape format with a ratio of 0.571 which is similar to the Morgan drawing's landscape ratio of 0.556 (32.5 × 58.5 cm.).^25^ Second, an 1831 auction catalogue lists a second Crucifixion painting by Benjamin West of unknown dimensions that was sold by his son for 45 Guineas.^26^ While the catalogue's description makes no mention of the painting's dimensions, its lengthy description does not associate the painting to St. George's Chapel which contrasts with the 1829 catalogue's description, thereby suggesting that the second painting listed by Galt was sold in 1831 (Table 1).

The location of both Crucifixion paintings remains a mystery.

## Funding

Open access was funded by a grant from the Wellcome Library Open Access Fund.

## Figures and Tables

**Figure. 6:**
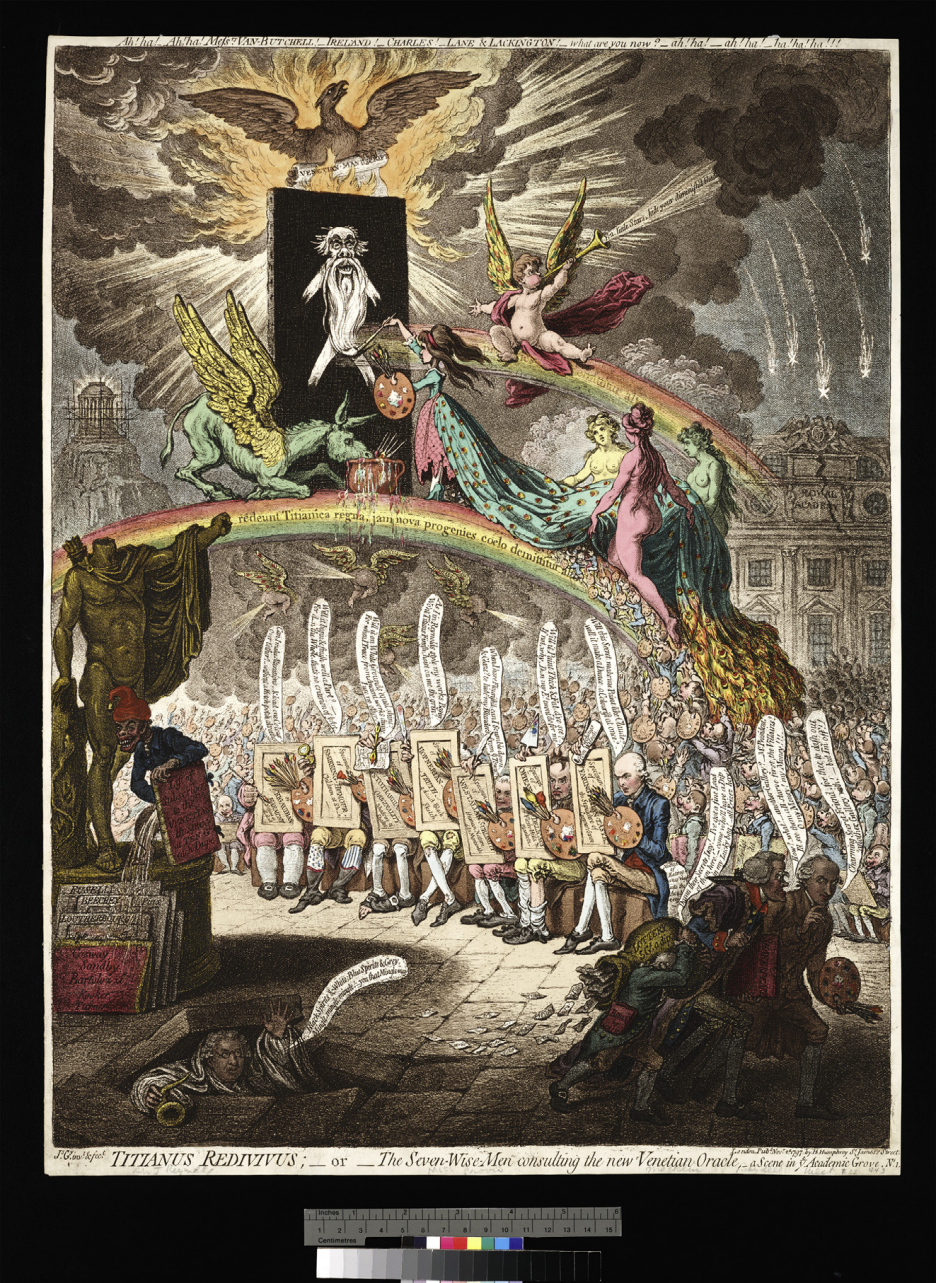
Titianus redivivus, or, The seven-wise-men consulting the new Venetian oracle [graphic]: a scene in [the] academic grove. No. 1/Js. Gy. invt. & fect. London: Pubd. Novr. 2d, 1797, by H. Humphrey, St. James's Street, [2 November 1797]. (Courtesy of The Lewis Walpole Library, Yale University) West is at the extreme right. Note the ape leaning on a volume titled “List of subscribers to the Venetian humbug at Ten G. each Dupe”. He is urinating on the portfolios of those academicians who were not fooled, which includes Cosway who witnessed Carpue's experiment. http://www.racollection.org.uk/ixbin/indexplus?record=ART309&_IXFILE_=templates/pages/kiosk/video3.html Nick Savage [Accessed July 21, 2015].

**Table 1:** Comparison of West's drawings and paintings of The Crucifixion.

	First Galt painting	Second Galt painting	Morgan drawing
Page in Galt where listed	217	219	Galt did not list drawings

Dimensions (Height × Width)	7.5 × 6 ft in Galt	16 × 28 ft in auction catalogue	32.5 × 58.5 cm in library catalogue

Ratio (Height × Width)	1.25	0.57	0.56

Date of last public display	1829	1831	2015

Provenance	Benjamin West (estate)	Raphael West	Estate of the artist; by descent; Harry Margary, the great-grandson of Maria West (daughter of Raphael West); Thos. Agnew and Sons, Ltd., London; from whom purchased 60 drawings, 17 June 1964, and 197 drawings, 15 February 1965, by M. Knoedler and Co., Inc.

Auction catalogue description	“*From this design* [emphasis added] a painted glass was to be executed for the large west window in St. George's Chapel, at Windsor.”	“George III … projected a chapel to be erected by Mr. James Wyatt, within the ancient palace of Windsor, which was to be enriched with paintings and sculpture.” No mention of West	

Outcome	Sold to a Mr. Ward or Word, price unknown	Purchaser unknown, Sold for 45 G.	Purchased by the Morgan Library in 1970
